# Personal Phone Calls Lead to Decreased Rates of Missed Appointments in an Adolescent/Young Adult Practice

**DOI:** 10.1097/pq9.0000000000000192

**Published:** 2019-07-29

**Authors:** Rebecca Penzias, Virginia Sanabia, Kyra M. Shreeve, Urmi Bhaumik, Caitlin Lenz, Elizabeth R. Woods, Sara F. Forman

**Affiliations:** From the * Division of Adolescent/Young Adult Medicine, Boston Children’s Hospital, Boston, MA; † Office of Community Health, Boston Children’s Hospital, Boston, MA; ‡ Department of Pediatrics, Boston Children’s Hospital, Boston, MA.

## Abstract

**Introduction:**

Nationally, hospital practice missed appointment rates are high. Our goal was to reduce the rate of missed appointments in an Adolescent/Young Adult Practice through quality improvement methods.

**Methods:**

During the 12-month intervention period, administrative staff called patients the day before their primary or specialty care appointments to remind them of the date, time, and location, as well as patients who did not attend their appointments to ask about the reason for their missed appointment. We implemented Plan-Do-Study-Act interventions and analyzed data to compare missed appointment rates between the 12 months before and after February 1, 2017, the project intervention date.

**Results:**

Results showed significant reductions in the missed appointment rate for the Adolescent/Young Adult Practice. A control chart showed a shift in the mean overall percent of completed appointments from 76.7% to 79.2%. The most common reasons for missed appointments included forgetting (39.2%), conflicts with work/school (11.0%), or emailing the provider without contacting administrative staff (7.8%). There were significant reductions in missed appointment rates for both males and females as well as patients who were ≥20 years old, identified English or Spanish as their primary language, had public or private insurance, identified as Black or Hispanic, or did or did not require an interpreter.

**Conclusion:**

These data show that targeted interventions such as personalized reminder calls can be effective in reducing patient missed appointment rates in Adolescent/Young Adult Practices.

## Abstract 2

Supplemental Digital Content is available in the text.

## INTRODUCTION

Nationally, missed appointment rates are highly variable, ranging from 5% to 55%.^1^ Certain patient populations have higher rates, which may contribute to reduced access to care. These vulnerable populations include those who have public insurance,^2^ do not speak English as a first language,^3^ or identify as racial/ethnic minorities.^4^ Adolescents/young adults have higher missed appointment rates than pediatric and adult patients. These higher rates may be due to challenges transitioning to treatment self-management. This population may also have difficulty getting to appointments, as many are not able to drive or do not own a car.^1,2^

Previous studies have examined the effects of appointment reminders on adolescents. One 1993 study in an adolescent clinic found that patients who received a reminder call had an attendance rate of 55.6% compared with 44.1% among those who did not.^5^ Another study within an adolescent clinic from 2002 found that reminder calls significantly reduced the rate of missed appointments from 20% to 8%.^6^ However, these studies were performed before the integration of cell phones throughout the adolescent population, and most youth did not have a phone.

More recently, research has been performed on missed appointment rates among adolescents; however, these studies focus on distinct subpopulations with high medical need, such as youth with type 2 diabetes mellitus or HIV.^7,8^ One study in an adolescent psychiatry clinic found that the missed appointment rate decreased by 4% through motivational interviewing.^9^ Other studies have utilized reminder message systems to reduce rates of missed appointments; however, these interventions took place in adult pain, adult primary care, community dental, and primarily adult gastroenterology clinics.^3,10–12^ These previous studies have indicated that personal reminder calls can significantly decrease missed appointment rates in various subpopulations, but there is little recent research focused on missed appointment rates of the overall adolescent population.

In addition to studying the efficacy of reminder calls to address the missed appointment rate, some research has focused on identifying reasons for missed appointments. Recent studies in pediatric neurology and pain clinics and adult primary care settings identified some of the most common reasons that patients miss their appointments, including forgetting, scheduling conflicts, and miscommunication.^4,13–15^ One study examined factors that contributed to missed appointments in a pediatric primary care clinic; however, this study used a convenience sample of caregivers.^16^ Identifying the reasons for why patient populations miss their appointments can provide insight into approaches for reducing the missed appointment rate.

This intervention aimed to reduce the overall rate of missed appointments in a northeast urban Adolescent/Young Adult Practice and thereby improve access to care, particularly for patients with higher missed appointment rates, using Quality Improvement (QI) methodology.^17,18^ This intervention is especially helpful for the adolescent/young adult population because it addresses unique challenges that adolescents face during the transition to independent care-management, including remembering and getting to appointments. Personal reminder calls may help to ensure that patients are aware of upcoming appointments and provide an opportunity to make transportation arrangements. Additionally, many adolescents have their parent/caregiver’s contact information listed when they first begin coming to the clinic, but these numbers are not always the best or most confidential method of contact as patients grow older. Therefore, collecting accurate phone numbers for adolescent patients themselves may allow the clinic to reach patients more reliably while promoting patient autonomy.^19^ Higher rates of kept appointments may lead to improved outcomes and overall health status, increased access to care, fewer avoidable Emergency Department (ED) visits and hospitalizations, and greater patient satisfaction.^20^

## METHODS

### Clinic Context

The Adolescent/Young Adult Practice in which we performed this study is a large multidisciplinary, primary and specialty care practice situated at an urban hospital in the northeast which provides medical, psychological, psychiatric, social work, and nutrition services for patients ages 12–25 years. The mean age of patients is 20.2, and 64.8% were females, 43.8% have public insurance, and 44.6% identify as Black, while 25.7% identify as Hispanic. Between February 1, 2017, and January 31, 2018, there were 14,985 total visits, with primary care visits making up 74.4% and specialty care visits making up the remaining 25.6%. Specialty care services include treatment related to eating disorders, reproductive endocrinology and long-acting reversible contraceptives, weight management, chronic fatigue and complex care, HIV testing/counseling, and media addiction. We included all primary and specialty care patients except for those with confidential appointments for mental health or HIV/STI testing/counseling.

### Intervention

A dual intervention was designed to reduce the missed appointment rate by (1) instituting personal reminder calls before appointments and (2) analyzing why patients missed their appointments. Practice administrative staff called patients of residents, fellows, and nurse practitioners the day before their appointments to remind them of the date, time, and location. If a patient or parent indicated that their appointment needed to be rescheduled, it was not counted as a missed appointment. Staff members already provided personal reminder calls for patients of attending physicians (~43% of the overall population) before this study, but the intervention, which included identifying the best recipients for reminder calls, applied to the entire clinic population. Thus, we included these patients in the analyses. Staff also attempted phone contact for all patients who had a missed appointment between February 1, 2017, and January 31, 2018, to assess their reason for missing their appointment by asking a set of questions (see Appendix A, available as Supplemental Digital Content at http://links.lww.com/PQ9/A109).

### Evaluation of the Intervention

The main outcome was the change in the missed appointment rate in the Adolescent/Young Adult Practice. To be included in analyses, patients had to have at least one kept or missed appointment between February 1, 2016, and January 31, 2017 (the year before intervention), and at least one kept or missed appointment between February 1, 2017, and January 31, 2018 (the intervention year). We excluded patients from analyses if they canceled their appointment or arrived but were not seen during the baseline or intervention periods. Patients who had confidential appointments for mental health or HIV/STI testing/counseling were also excluded.

The primary process measure was the percentage of reminder calls in which staff reached the patient. We performed Plan-Do-Study-Act cycles throughout the intervention, which involved analyzing the effects of the intervention, identifying potential challenges through root cause analysis, and implementing additional changes. As a part of these Plan-Do-Study-Act cycles, 2 additional interventions were added, including the integration of a checklist into the appointment check-in process to standardize the collection of contact information and the adjustment of the time of reminder calls from morning (9–11 am) to late morning/afternoon (11 am–3 pm).

Individual- and population-level hospital administrative data, including demographic information and the number of scheduled and missed appointments, was collected in the Adolescent/Young Adult Practice between February 1, 2016, and January 31, 2017. We compared these data to the number of scheduled and missed appointments in the same practice between February 1, 2017, and January 31, 2018.

Patients contacted by staff regarding their missed appointment were informed that their answers were voluntary and confidential. Staff entered data into Microsoft Excel 2010 (Microsoft, Redmond, Washington, US) and REDCap version 8.9.2 (Vanderbilt University, Nashville, Tennessee, US).^21^ The reasons why patients missed their appointments were divided into categories as well as analyzed in aggregate. We conducted root cause analysis to identify potential reasons for missed appointments based on the responses given by patients. Five categories of root causes were determined based on the most common responses during a pilot period over the 2 weeks before the intervention start date. Subsequent responses during the intervention period were categorized by type.

This QI project did not require prospective IRB review because the aims were to decrease the practice’s missed appointment rate and improve access to care through QI interventions. However, IRB approval for access to QI data and waiver of the need for informed consent was obtained.

### Population-Level Analyses

We plotted the percentage of completed appointments before and after the intervention start date as a p prime control chart with 3 SDs around the mean. The centerline was shifted when 8 consecutive points fell above the center line, and there was a baseline of at least 12 points.^22^

### Individual-Level Analyses

The results observed at the population level were verified through analysis of patients before and after the intervention start date. Paired *t*-tests (two-way ANCOVA) were performed in SPSS using patient demographics from administrative data, including dichotomous variables such as age (<20 versus ≥20), sex (male versus female), insurance type (public versus private), language (English versus Spanish), and interpreter needed (yes versus no), and categorical variables such as race/ethnicity, appointment time, and appointment day of the week. We also compared data points for the clinic’s primary, specialty, and overall patient populations using risk ratios.

## RESULTS

### Population-Level Results

**Fig. 1.**
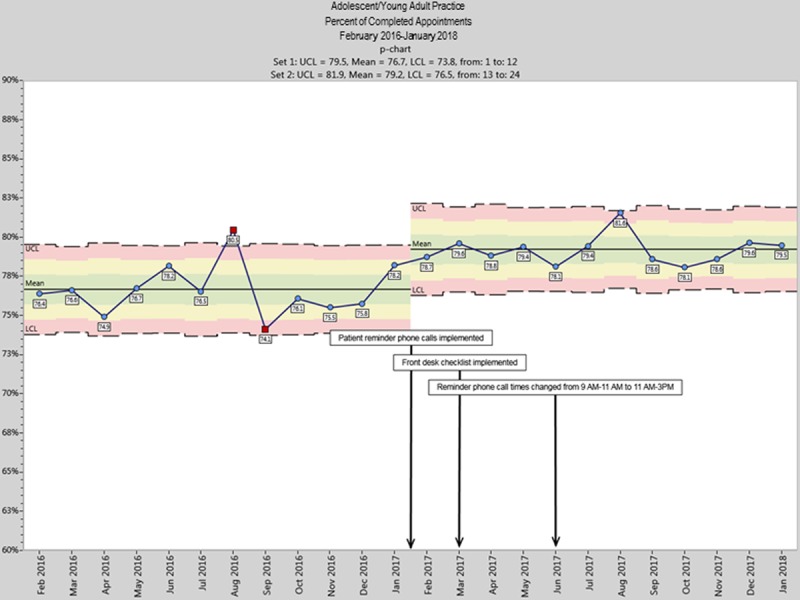
Control chart of the percent of all completed appointments in the Adolescent/Young Adult Practice from 1 year before the intervention (February 1, 2016–January 31, 2017) and 1 year after implementation of the intervention on February 1, 2017 (February 1, 2017–January 31, 2018). The intervention line was drawn at the start of the pilot period (January 19, 2017), since those calls appeared to shift the mean. UCL indicates upper confidence limit; LCL, lower confidence limit.

During the intervention year, 24,292 appointments were scheduled. Population-level analyses of completed appointments show a significant increase in overall appointment completion rate from 76.5% (lower confidence limit = 73.7; upper confidence limit = 79.4%) to 79.1% (lower confidence limit = 76.4; upper confidence limit = 81.8%). Figure 1 is a p prime control chart annotated with 3 process changes: (1) implementation of reminder calls (including pilot calls beginning January 19, 2017, since they appeared to initiate the mean shift); (2) implementation of a front desk checklist to standardize collection of accurate contact information; and (3) change in reminder call times from morning (9–11 am) to late morning/afternoon (11 am–3 pm) when calls were more likely to be answered. The second and third process changes did not appear to make additional improvements after the implementation of personal reminder calls.

### Individual-Level Results

Individual-level analyses revealed a statistically significant decrease in missed appointment rate for all patients from 23.3% to 20.8% (*P* < 0.001). The missed appointment rate decreased from 25.0% to 22.4% (*P* < 0.001) and from 14.7% to 13.1% (*P* = 0.04) among primary and specialty care patients, respectively. Specific groups of patients had significant reductions in missed appointment rates, including patients who had public (27.6%–25.2%, *P* < 0.001) or private (16.2% to 14.2%, *P* < 0.001) insurance, were males (26.1% to 22.1%, *P* < 0.001) or females (22.4% to 20.4%, *P* < 0.001), identified as Black (31.4% to 27.8%, *P* < 0.001) or Hispanic (28.0% to 26.0%, *P* = 0.01), were 20 years or older (26.8% to 23.7%, *P* < 0.001), whose primary language was English (23.1% to 20.5%, *P* < 0.001) or Spanish (27.2% to 24.1%, *P* = 0.008) and who did (25.2% to 22.5%, *P* = 0.03) and did not need an interpreter (23.3% to 20.6%, *P* < 0.001) (Table 1). Significant reductions were not observed for patients who identified as White or were <20 years old.

**Table 1.**
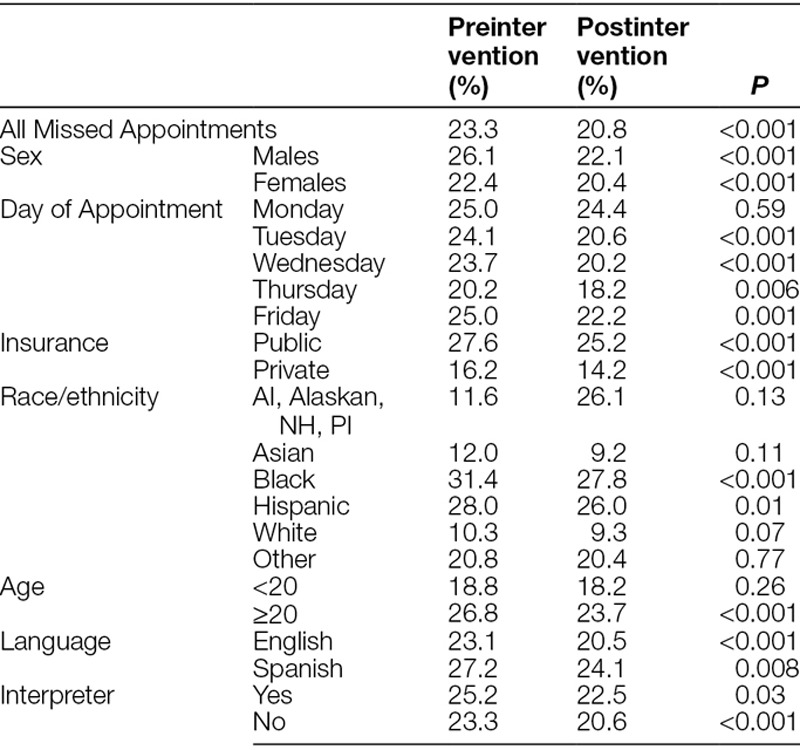
Patient Missed Appointment Rate for All Scheduled Appointments 1 Year Before (n = 23,597) and 1 Year Following (n = 24,292) the February 1, 2017 Intervention Start Date

The missed appointment rates between primary and specialty care visits reveal that patients who have primary care appointments are 1.71 times (*P* < 0.001) more likely to miss their appointment compared with specialty care patients (Table 2). For both primary (*P* < 0.001) and specialty care (*P* = 0.04), patients were 1.12 times more likely to miss preintervention appointments compared with postintervention. Overall, patients had positive responses to the personal reminder calls.

**Table 2.**
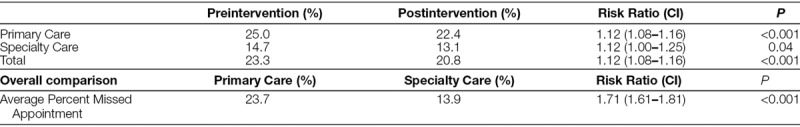
Percentage of Missed Appointments by Primary or Specialty Care Before (n = 23,597) and After (n = 24,292) Intervention and Comparison of Overall Missed Appointment Rates

### Reasons Why Patients Missed Their Appointments

**Fig. 2.**
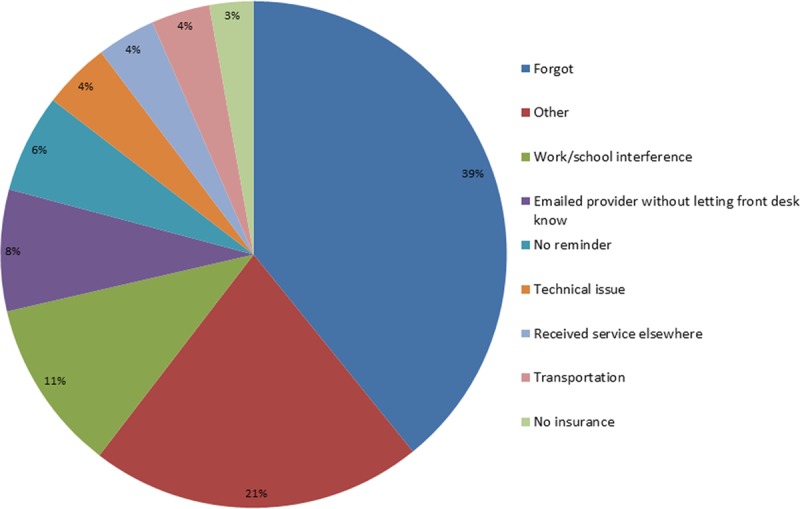
Reasons why patients did not show up for their appointments (n = 748).

We contacted 748 patients or parents of patients who missed their appointments (14.8% response rate). Figure 2 shows the reasons given for missed appointments. The majority of patients forgot (39.2%), had work or school (11.0%), or emailed their provider without contacting administrative staff (7.8%). Some patients did not receive a reminder call (6.3%), had an issue with canceling their appointment (4.3%), had transportation (3.7%) or insurance (2.8%) issues, or received service elsewhere (3.7%). We combined all other reasons into an “other” category (21.3%). This category includes patients who arrived very late or overslept (2.1%), had emergencies or hospital admissions (1.2%), were sick (0.8%), or declined to come (0.7%).

### Root Cause Analysis

**Fig. 3.**
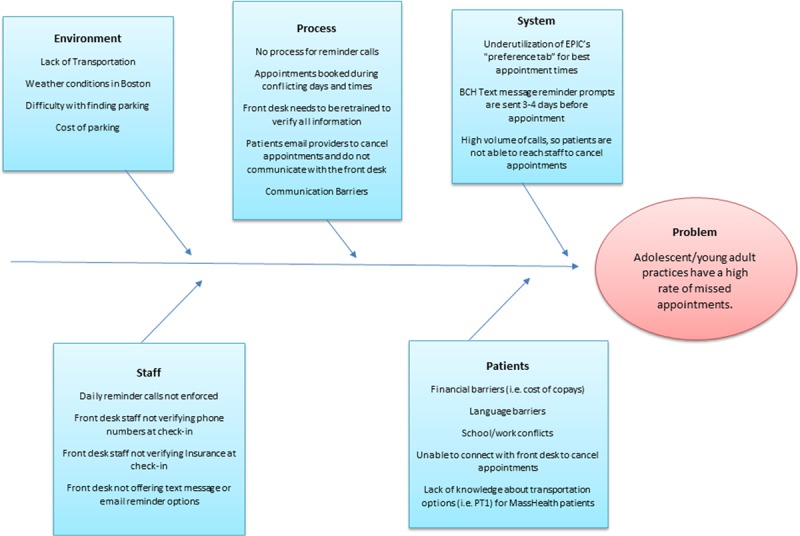
Fishbone (Ishikawa) diagram depicting potential root causes of the Adolescent/Young Adult Practice missed appointment rate, including environment, process, system, staff, and patient causes.

We identified and categorized the potential root causes of the missed appointment rate problem by type using a fishbone (Ishikawa) diagram^17^ (Fig. 3). Causes were divided into 5 categories: environment, process, system, staff, and patient.

## DISCUSSION

These data show that personalized reminder calls were effective in reducing the missed appointment rate for the Adolescent/Young Adult Practice.^2–4^ These data reveal disparities in missed appointment rates for vulnerable populations, such as racial/ethnic minorities. Personalized reminder calls were particularly effective at improving kept appointment rates for specific populations, including patients who identify as Black or Hispanic or are ≥20 years. The intervention was effective for patients regardless of gender (male or female), primary language (English or Spanish), and need for an interpreter. We did not observe significant reductions for patients who identified as White or were <20.

We observed a 2.5% improvement in the missed appointment rate. Earlier studies that evaluated the use of reminder calls in adolescent clinics found larger improvements in missed appointment rates; however, these were conducted before the introduction of cell phones as a major method of communication, which has provided clinics with the option to contact adolescent patients directly. Prior studies may have had higher rates of improvement because parents whose phone numbers were contacted might have been more likely to make sure that their child attended the appointment. Families already received automatic calls 2–3 days before the appointment, so the addition of personal calls and accurate listing of preferred numbers may have added to the improvement. Many adolescents requested that personal reminders go to themselves, leaving the responsibilities of remembering and attending appointments to them. This request may be why the greatest improvement was observed among patients ≥20 years since they were better equipped to manage their appointments. A recent intervention conducted in an urban, culturally diverse family medicine clinic saw a similar reduction of 3% in their missed appointment rate. However, this intervention targeted only a small, high-risk, primarily adult population.^23^ Other studies showed reduced missed appointment rates after interventions, but they were in adult pain and primarily adult gastroenterology clinics.^3,12^ The decrease in missed appointment rate observed in this study was comparable to recent studies that have implemented similar interventions, but because there are many psychosocial contributors to missed appointments in the adolescent/young adult population, this clinically significant degree of improvement was greater than expected. The pilot in the last 2 weeks of January 2017 probably initiated the mean shift before the intervention period, but this shift is sustained throughout the intervention.

The main intervention was the implementation of personalized reminder calls. This intervention aimed to reduce the missed appointment rate by ensuring that patients were aware of upcoming appointments and had the opportunity to cancel if necessary. Root cause analysis was used to determine patients’ reasons for missed appointments to identify additional potential actions to address the issue. In response to the root cause analysis, we shifted the timing of calls from morning (9–11 am) to late morning/afternoon (11 am–3 pm), when patients were more likely available. Additionally, we added a standardized checklist to the front desk check-in process to confirm the correct contact information for patients, which may have made it more likely for staff to reach them with reminder calls for subsequent appointments. Though we observed no additional significant improvement after implementing the 2 secondary interventions, they might have helped sustain the improvement throughout the study year.

Compared with other days of the week, patients who had appointments on Monday did not have a significant change in the missed appointment rate with reminder calls on Friday. Reminder calls placed on Friday may not have been as effective because they occurred 3 days before the appointment compared to other calls, which were placed the day before.

Since we observed a decrease in missed appointments, we performed a rough cost analysis to determine whether the decrease was sufficient to be worthwhile given the time required to make the personal calls. Assuming that reminder calls take about 2 minutes, and staff can complete 30 calls in 60 minutes, the 16,000 annual visits in the clinic would require 533 hours of labor. At the cost of $17/h and 31% benefits for an administrative representative to place reminder calls, this amounts to ~$11,870 of annual labor costs for ~400 more visits. In addition to improving care and access, the costs of labor could lead to cost savings if improved access increases visits to the medical home, reduces ED visits and hospitalizations or other costly health interventions, and enables patients to receive preventive visits. Due to limited access to health outcome data, it is difficult to determine if the intervention was cost effective. However, although the number of payments for the 400 additional visits were not available, they likely exceeded the expenses. The overall feedback from individuals who received calls was positive, but further evaluation is needed to determine the impact of the calls on patient experience.

There are some limitations. We piloted this intervention in one urban Adolescent/Young Adult Practice in the northeast, and the results may not be entirely generalizable to other populations/areas. Other unidentified clinical changes during the same period may have had an impact on increasing the kept appointment rate. Nonclinical factors, such as construction and parking availability on the medical campus or changes in weather, may also have affected the kept appointment rate. Additionally, the patients who had confidential appointments, including appointments for mental health or HIV/STI testing/counseling, were excluded from analysis and therefore the missed appointment rate does not reflect the entire patient population. Despite these limitations, this study indicates that personal reminder calls can be effective for decreasing the rate of missed appointments among adolescents.

## CONCLUSION

Personalized reminder calls can be an effective way to improve kept appointment rates in an urban adolescent/young adult primary and specialty care practice. Future studies should link health outcomes, such as ED visits and hospitalizations, with appointment rate data to see if increasing the kept appointment rate is an effective way to reduce the number of ED visits and hospitalizations. Ultimately decreasing the rate of missed appointments may reduce costs and increase access to care. It may also improve quality of care by allowing a greater number of patients to receive essential screenings and treatment for conditions commonly found among adolescents, such as sexually transmitted infections or depression.^24,25^

## ACKNOWLEDGEMENTS

Assistance with the study: We would like to thank Danielle McPeak, BA, for her assistance in obtaining overall clinic data.

## Supplementary Material
