# Supplementary figures and images for: Personal Phone Calls Lead to Decreased Rates of Missed Appointments in an Adolescent/Young Adult Practice
Source: Pediatr Qual Saf. 2019 Jul 29;4(4):e192. doi: 10.1097/pq9.0000000000000192 (PMC6708648; doi:10.1097/pq9.0000000000000192)

**Appendix A:** Questionnaire used to assess patients’ reasons for missed appointments


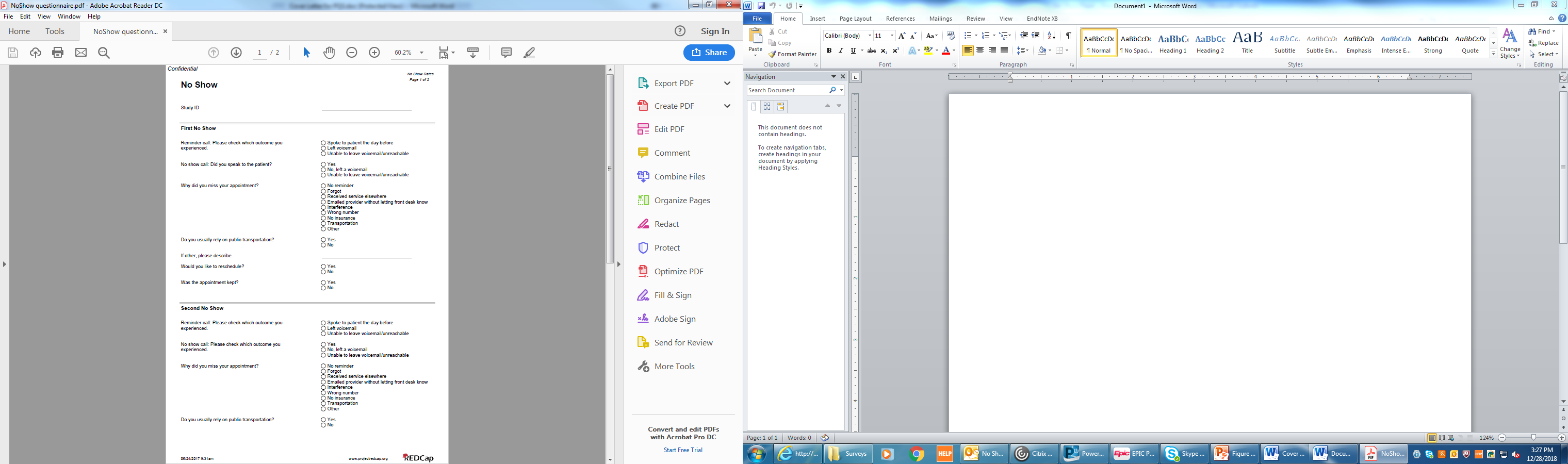

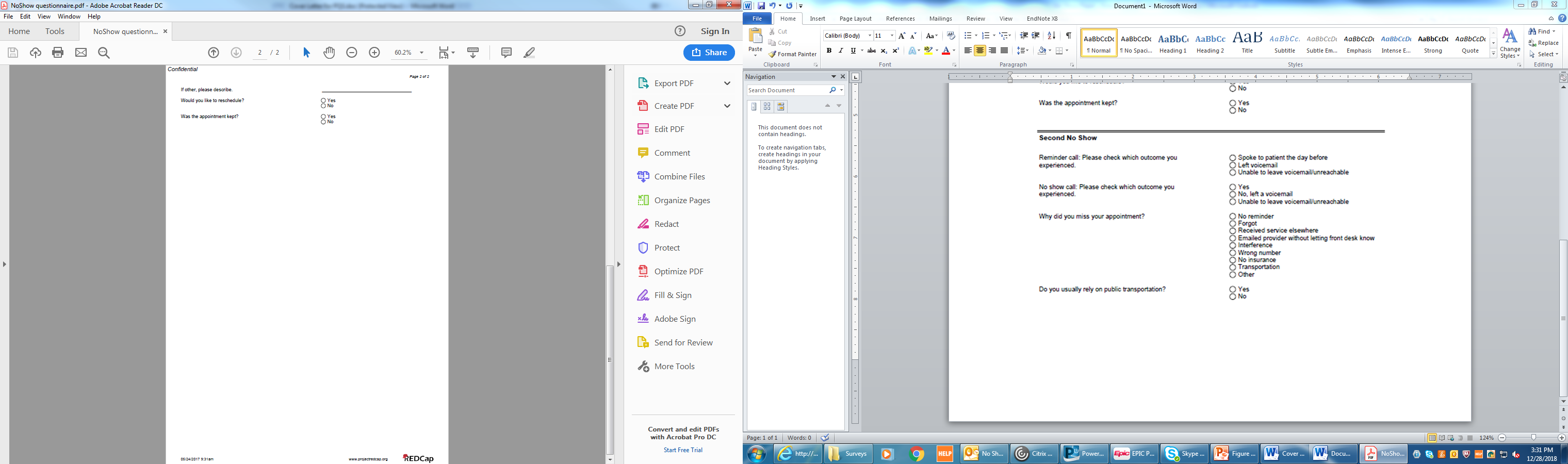

Supplement: Supplementary file 1 [file pqs-4-e192-s001.docx]
